# Contrasting Effects of Maternal and Paternal Age on Offspring Intelligence: The clock ticks for men too

**DOI:** 10.1371/journal.pmed.1000042

**Published:** 2009-03-10

**Authors:** Mary Cannon

## Abstract

Mary Cannon discusses the implications of a new cohort study showing an association between increasing paternal age and poorer cognitive abilities in the offspring.

Both maternal and paternal ages are increasing in the developed world. The average age of mothers at time of childbirth has increased from 26.4 years in 1974 to 29.3 years in 2002, while the average age of fathers has increased from 29.2 years in 1980 to 32.1 years in 2002 [1]. This increase in the average parental age is most likely due to the societal trend for couples to delay starting a family for career or financial reasons. The concept of the female “biological clock” (the effect of increasing maternal age on reducing fertility) is well known and is a source of anxiety for many women [2]. In contrast, the consequences of increasing paternal age on fertility and other adverse reproductive outcomes are rarely discussed [3].

## Effects of Paternal Age on Offspring Outcomes

Evidence is accumulating that advanced paternal age may exhibit a wider range of effects on the health and development of the offspring than increased maternal age (which is largely confined to risk for Down syndrome). Advanced paternal age is a risk factor for childhood conditions such as cleft lip and palate; childhood cancers and congenital heart defects [1]; and neuropsychiatric conditions such as autism [4], schizophrenia [5,6], epilepsy [7], and bipolar disorder [8]. Advanced paternal age also appears to affect mortality, and an intriguing analysis of family history data from European nobility found that older age of fatherhood (greater than 45 years) is associated with a reduction of about two years in the life span of daughters [9].

**Linked Research Article**

This Perspective discusses the following new study published in *PLoS Medicine*:

Saha S, Barnett AG, Foldi C, Burne TH, Eyles DW, et al. (2009) Advanced paternal age is associated with impaired neurocognitive outcomes during infancy and childhood. PLoS Med 6(3): e1000040. doi:10.1371/journal.pmed.1000040

Using a sample of children from the US Collaborative Perinatal Project, John McGrath and colleagues show that the offspring of older fathers exhibit subtle impairments on tests of neurocognitive ability during infancy and childhood.

Some of these associations (notably that for schizophrenia) are more extensively replicated than others, but the body of evidence implicating paternal age as a risk factor for a range of adverse offspring outcomes should not be ignored. What is the postulated mechanism for these associations?

## Putative Genetic Mechanisms of Paternal Age Effect

Most commentators attribute these associations to some form of genetic effect, with the greatest consensus in favour of spontaneous mutation. Genomic studies show that sperm cells undergo more mutations than ova during the life span [10]. Thus delaying fatherhood might contribute to an increased incidence of mutations that can give rise to developmental and neuropsychiatric disorders in the population. Epigenetic mechanisms, such as hypermethylation, increase with age and may be an alternative explanation [11,12].

## Intermediate Phenotypes

Rather than a direct genetic effect, paternal age could increase risk for a range of neuropsychiatric outcomes in an indirect manner by increasing the likelihood of an “at-risk” or precursor phenotype in offspring. Support for this hypothesis comes from a study by Weiser and colleagues, who analysed data from an Israeli cohort of 10,000 male conscripts and found that offspring of both very young fathers (less than 20 years) and older fathers (greater than 45 years) had impaired social function [13]. Sons of older mothers (greater than 40 years) also had poorer social function. Poor social function has been shown to be a precursor for many psychiatric disorders, such as schizophrenia [14].

## Parental Age and Intelligence of Offspring

A new study by John McGrath and colleagues in this issue of *PLoS Medicine* examines the association between paternal and maternal age and impaired neurocognitive ability in childhood (another putative intermediate phenotype) [15]. The authors use data from the Collaborative Perinatal Project, a large birth cohort of more than 50,000 individuals born between 1959 and 1965 in 12 centres in the United States, who were followed up throughout childhood. Cognitive measures were collected at three time points: eight months, four years, and seven years. The use of a cohort from the 1960s means that the association between parental age and offspring intelligence is largely unconfounded by the possible neurocognitive effects of assisted reproductive technology (which began in 1978) or the possible psychosocial effects of complex (or blended) stepfamily structures, which have become more common over the past decade.

McGrath and colleagues show remarkable contrasting effects of paternal and maternal age on the cognitive abilities of the offspring [15]. Increasing maternal age is associated with *superior* performance on intelligence tests in a linear fashion whereas increasing paternal age is associated with significantly *poorer* performance on five out of six of the measures tested.

A second notable aspect of this study is the effect of adjustment for socio-economic factors. Controlling for parental mental health and socio-economic status, measured using a composite score that indexes maternal and paternal education as well as family income, resulted in a marked attenuation of the effect of both maternal and paternal age on the intelligence scores. For instance, the average difference in IQ between the offspring of a father aged 20 and a father aged 50 decreases from six points to three points after adjustment for socio-economic factors.

These intriguing findings give rise to two questions: (1) Why should the offspring of older fathers, but not older mothers, have poorer performance on intelligence tests? and (2) If genetic effects are responsible, then what role do social factors play?

## The Role of Social Factors

Social advantage (in the form of economic security and increased education) may compensate to a certain extent for the biological risks in delaying motherhood [16]. McGrath and colleagues find that delayed fatherhood does not appear to convey this social advantage in the form of better cognitive test scores [15]. Is this due to some inherent difference in the way in which older fathers and older mothers interact with their children? Or is this due to spontaneous mutation—bearing in mind that studies in rodents show that paternal age significantly influences developmental and behavioural outcomes in offspring [12]? Of course, both effects could be operating in conjunction in humans. For instance, Reichenberg and colleagues have postulated that the incidence of genetic mutations may be influenced by age at fatherhood, which in turn may be influenced by the socio-cultural environment or by personality characteristics [4].

## Conclusion

McGrath and colleagues show the importance of taking socio-economic factors into account when examining the issue of paternal age [15]. Could the paternal age effect on offspring intelligence be due to so-called residual confounding, whereby adjustment does not fully remove the effect of a confounder [17]? In other words, if we could adjust the association for every relevant socio-economic and interpersonal variable (both known and unknown) using precise measures, then perhaps we could eliminate the effect of paternal age on intelligence completely. New explanatory models are needed that can encompass socio-cultural and interpersonal factors as well as biological variables. Perhaps then we can decide when is the best time to be a mother…or father.
